# Prediction of the Low-Velocity Collision Response Characterization of a Plate Structure Considering the Strain Hardening Effect

**DOI:** 10.3390/ma18133040

**Published:** 2025-06-26

**Authors:** Xin Xiao, Xiaochun Yin, Huaiping Ding

**Affiliations:** 1 School of Finance and Mathematics, Huainan Normal University, Huainan 232038, China; xiaoxin@hnnu.edu.cn; 2 School of Physics, Nanjing University of Science and Technology, Nanjing 210094, China; yinxiaochun@njust.edu.cn

**Keywords:** collision, plate, characterization, response case, collision force

## Abstract

The prediction of the low-velocity collision response of a plate has substantial engineering significance. This paper presents a study to predict the low-velocity collision response characterization of a plate considering the strain hardening effect struck by a rigid sphere. To investigate the efficiency of the collision characterization diagram (CCD) based on the theoretical contact stiffness in characterizing the collision response case and calculating the maximum collision force, the intensive collision cases considering the strain hardening effect are implemented by the finite element (FE) method. It shows that CCD is inaccurate for the collision cases, considering the strain hardening effect. To modify CCD, a new contact stiffness is proposed to replace the theoretical contact stiffness. A universal analytical expression of the new contact stiffness is presented according to the intensive FE simulations for a wide range of materials of a plate, considering the strain hardening effect. A modified collision characterization diagram (MCCD) is then proposed by using the new contact stiffness, which makes up for the deficiencies of CCD. MCCD is validated by the FE simulations with different collision energies, plate materials, and structural constraints. The results show that MCCD can accurately and quickly predict the response case and the maximum collision force.

## 1. Introduction

The elastic–plastic collisions are extremely extensive and rather complicated in various engineering fields [1,2,3,4,5]. Therefore, the precise prediction of the low-velocity collision response of an elastic–plastic plate has substantial engineering significance. Common metal material is one of the common elastic–plastic materials, which is easy to harden. The extensive investigations have been implemented on the collisions of the metal material with the analytical, experimental, and numerical methods without considering the strain hardening effect [6,7,8,9,10]. The same attention needs to be paid to the study of the low-velocity collision response of a plate, considering the strain hardening effect as well.

For the low-velocity collisions of plates, the target plates are usually considered to be the elastic-perfectly plastic [7,8,11]. For the elastic-perfectly plastic materials of low-velocity collisions, researchers show that there are the following cases of responses categorized by the collision mass, i.e., the half space case, the infinite response case, the intermediate case, and the quasi-static case [12,13]. The half-space case is approximated as a very light sphere striking a completely hard plate [14], and this case can be solved by the local collision on the half-space structure [15,16]. The infinite response case is approximated as a relatively light sphere striking a normal plate [12], and the response of this collision is only controlled by deformation waves [17]. Hence, the infinite response case is also called the wave-controlled case, and it includes the half-space case. The quasi-static case is approximate as a relatively heavy sphere striking a normal plate [15], and the plate behaves like a static structure subjected to external loads [16]. The intermediate case is between the infinite response and quasi-static cases and is approximate as a little heavy sphere striking a normal plate [15]. The responses of this collision are very complicated [12] and may lead to complex or unpredictable collision responses [15,16].

According to these response cases of plate collisions, corresponding models can be used to analyze the histories of the collision force and other overall responses [18,19,20]. The models to response cases can be divided into three types: the infinite plate model, the quasi-static plate model, and the complete model [12,13]. The infinite plate model is applied for the infinite response case [21], the quasi-static plate model is used for the quasi-static case [22,23,24], and the complete model is suitable for the intermediate case [15,16]. For the infinite plate model and the quasi-static plate model, the histories of the collision force can be solved by the spring-mass models [21]. For the complete model, the complex derivations of expressions are required, or the complete FE models can be built for simulations [25,26,27,28,29].

These models are widely applied, efficient, and easy to use but have strict applicable limitations of ranges [12]. To solve the problem of the limitations, a collision characterization diagram (CCD) [16] was developed for the low-velocity collision response of elastic–plastic plates to predict the cases of collision responses, including the infinite response case, the intermediate case, and the quasi-static case. CCD was further applied to indicate the relationship of the maximum dimensionless collision force ${\bar{F}}_{max}$ and two dimensionless parameters, i.e., the relative mobility ζ and the relative stiffness λ [20]. The accuracy of CCD was verified by 17 collision experiments for the composite plates struck by the steel spheres [30]. Furthermore, the availability of CCD was numerically validated by 33 FE simulations for the metal plates struck by different rigid spheres [20].

For the low-velocity collision response characterization of the plate considering the strain hardening effect, the collision responses are more complicated, and few researches have been conducted [31,32,33,34,35]. In order to make use of CCD to predict the collision response case and calculate the maximum collision force, the responses of collisions on the plates considering the strain hardening effect should be deeply investigated and analyzed in detail.

Hence, the precise predictions of the low-velocity collision response characterization of the plate considering the strain hardening effect have an important influence on the substantial engineering significance. In this paper, the characterization of the low-velocity collision response of a plate considering the strain hardening effect struck by a rigid sphere is investigated by intensive FE simulations. In Section 2, the accuracy of CCD is investigated by the collisions considering the strain hardening effect based on intensive FE simulations. In Section 3, CCD is modified by using a new proposed $K_{n}$ method to replace the theoretical contact stiffness $K_{p}$, and the prediction accuracy of the modified collision characterization diagram (MCCD) is validated by a large amount of data. Finally, Section 4 concludes the studies.

## 2. Effect of Strain Hardening on the CCD

### 2.1. Collision Characterization Diagram (CCD)

**Figure 1:**
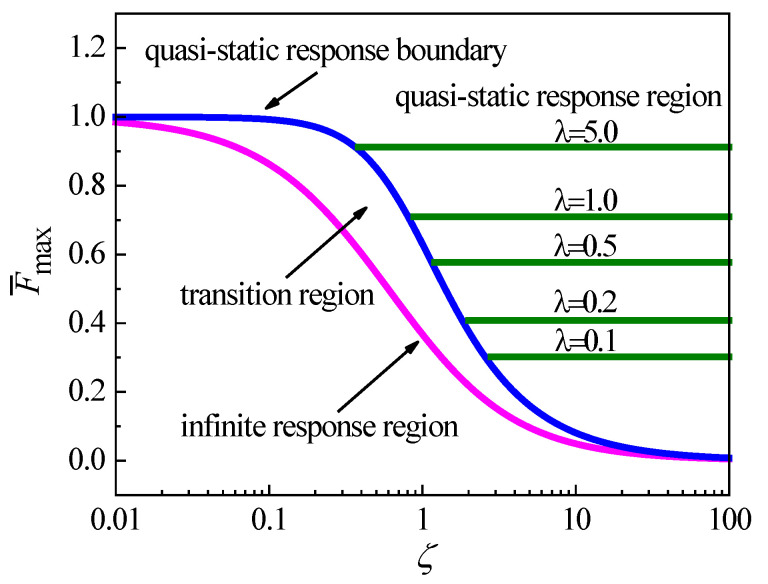
Collision characterization diagram (CCD).

A method called collision characterization diagram (CCD) [16,20] was proposed to indicate the relationship between the maximum dimensionless collision force ${\bar{F}}_{max}$ and two dimensionless parameters, i.e., the relative mobility ζ and the relative stiffness λ, as shown in Figure 1. CCD can be used to predict the collision response case and calculate the maximum collision force ${\bar{F}}_{max}$ to the collisions on the elastic–plastic plates.

In the CCD shown in Figure 1, the magenta curve is solved by the limiting solution of the spring-mass model [16,20] and represents the infinite response region. The blue curve is solved by the Swanson criterion [19] and represents the boundary of the quasi-static response. The region between the infinite response region and quasi-static response boundary is the transition region [16,20]. To the right of the quasi-static response boundary is the quasi-static response region, which includes a series of olive lines for various values of relative stiffness λ. By CCD, the collision response case can be predicted. If the relative mobility ζ is located in the curve of the infinite response region, the collision is characterized as the infinite response case. If ζ is over the quasi-static response boundary, the collision is characterized as the quasi-static response case. If ζ is located in the transition region, the collision is characterized as the intermediate response case. CCD can also be applied to calculate the maximum collision force $F_{max}$. For a given collision with the representative point (ζ, λ), $F_{max}$ can be directly calculated for two cases of infinite response and quasi-static. For the intermediate case, CCD can obtain the range of the maximum collision force $F_{max}$.

In CCD, the dimensionless maximum collision force ${\bar{F}}_{max}$ can be calculated by two dimensionless parameters [16,20], i.e., the relative mobility ζ and relative stiffness λ,

(1) $${\bar{F}}_{max}=\frac{F_{max}}{\sqrt{2E_{0}K_{p}}},\;\zeta =\frac{\sqrt{m_{s}K_{p}}}{2c},\;\lambda =\frac{K_{st}}{K_{p}},$$

where $F_{max}$, $\;E_{0}$, $m_{s}$, $K_{p}$, c, and $K_{st}$ are the maximum collision force, the collision energy, the collision mass, the theoretical contact stiffness, the structural impedance, and the plate static structural stiffness, respectively. The theoretical contact stiffness $K_{p}$ is solved according to the theoretical contact law [20]. For metals,

(2) $$K_{p}=2\pi Rp_{0},$$

where R is the radius of the sphere and $p_{0}=2.8\sigma_{Y}$, in which $\sigma_{Y}$ is the yield stress of plate.

**Figure 2:**
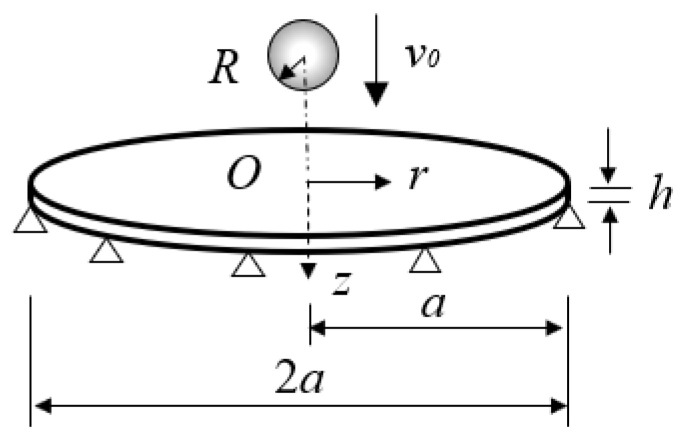
A simply-supported circular plate stuck by a sphere [36].

In this paper, the collisions between the simply-supported circular plate and the sphere are used to investigate the applicability of CCD considering the strain hardening effect by the finite element (FE) method established by the FE code LS-DYNA. Figure 2 shows a circular plate of the simply-supported constraint with the radius a and the thickness h struck by a sphere with the radius R, the mass $m_{s}$, and the collision velocity $v_{0}$ at the center of the plate. The dimensions and material properties of the plate and sphere are shown in Table 1, where ρ is the plate density and $\sigma_{Y}$, E, ν are the yield stress, elastic modulus, Poisson’s ratio of the plate, and n is the strain hardening exponent of the plate. To improve the computational efficiency, the material properties of the sphere are selected to be rigid. To conduct the intensive simulations ranging from small to large relative mobility ζ and relative stiffness λ, the plate radius a, the plate thickness h, and sphere mass $m_{s}$ are varied. The collision velocity $v_{0}$ is selected to be 1 m/s.

**Figure 3:**
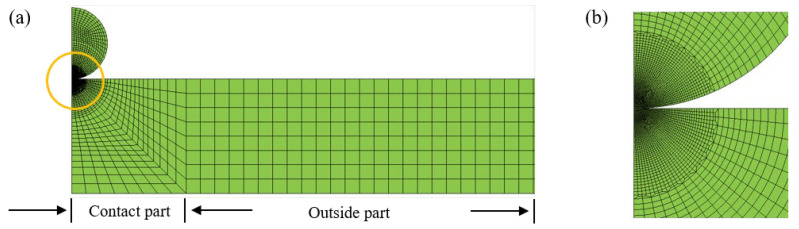
One FE model: (**a**) complete model, (**b**) contact part.

One example of the FE models is shown in Figure 3. The modeling method is similar to that in our previous paper [36,37], and it is not discussed here in detailed. The FE model consists of two parts: a contact part and an outside part. The contact part is divided into the contact region, connecting region and distant region. The number meshing the contact region is 80, according to the convergent tests [36], in order to densely and regularly simulate the contact deformation, as shown in Figure 3b. The distant region is meshed by the coarse but regular meshes are shown in Figure 3a. The mesh size of the connecting region increases gradually as shown in Figure 3a. The contact between the sphere and plate is selected to be surface to surface contact, and the friction between the contact surfaces is neglected [36,37]. The accuracy of the FE model is validated by the Hertz analytical solution and an elastic–plastic impact test [36].

In order to calculate two dimensionless parameters, ζ and λ, in CCD, the static structural stiffness $K_{st}$ and the structural impedance c are expressed by [16,20]

(3) $$K_{st}=\frac{16\pi \left(1+\nu \right)D}{\left(3+\nu \right)a^{2}},c=8\sqrt{\rho hD},$$

where $D=Eh^{3}/12(1-\nu )$ is the bending stiffness for the metal plates.

The applicability of CCD has been validated by few collision experiments [30] and FE simulations [20]. These situations are only suitable for a elastic–plastic plate struck by hard or rigid mass. CCD has been extended for complex collision situations under low collision energy [36] and moderate collision energy [37]. These studies are all based on the collisions between elastic-perfectly plastic materials. However, whether CCD is suitable for the characterization of the low-velocity collision response considering the strain hardening effect is required to be investigated.

### 2.2. Finite Element Simulations

To investigate the effect of the strain hardening for CCD, five values of strain hardening exponent, n= 0.1, 0.2, 0.3, 0.4 and 0.5, are selected. Different radii and thicknesses of the plate are chosen for every value of n to obtain the relative stiffness λ= 0.3. Sixteen masses of the sphere are selected for λ= 0.3 to vary the relative mobility ζ as shown in Table 2. The material properties of the plate are listed in Table 1, and the material properties of the sphere are rigid. The collision responses of the plate for 80 collision situations are simulated by the FE models mentioned in Section 2.1.

**Figure 4:**
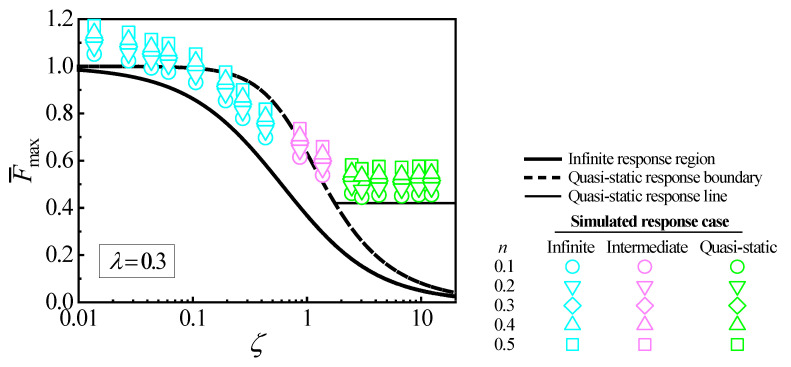
Simulated collision situations in CCD for five values of n.

Figure 4 shows a CCD for λ= 0.3, where the infinite response region and quasi-static response boundary are the same as shown in Figure 1. The quasi-static response line for λ= 0.3 in the quasi-static response region is constructed as the same method shown in Figure 1. To characterize the case of the collision response and calculate the maximum collision force of CCD, the maximum collision force $F_{max}$ for one collision situation is obtained from one FE simulation. The dimensionless maximum force ${\bar{F}}_{max}$ and the relative mobility ζ are solved using Equation (1). Each simulated collision situation can be represented by a point (ζ, ${\bar{F}}_{max}$) in CCD. The cases of collision responses can be characterized by time $t_{c}$ of the wave propagation from the collision position to the plate boundary [36]. The time $t_{c}$ is calculated by the bending wave speed $c_{L}$, $t_{c}=L_{c}/c_{L}$, where $L_{c}$ is the length of the closest distance from the collision position to the plate boundary. $c_{L}$ is

(4) $$c_{L}={\left(\frac{D}{\rho h}\right)}^{1/4}\sqrt{\omega },$$

where ω is the angular frequency of the plate. For the simply-supported circular plate,

(5) $$\omega =\frac{7.727}{a^{2}}\sqrt{\frac{\left(1+\nu \right)D}{\left(3+\nu \right)\rho h}}.$$

The cases of the collision responses are characterized by the ratio k of the total collision duration t to $t_{c}$. If k is less than 1.0, the response is characterized as the infinite response case. If k is larger than 10.0, the response is characterized as the quasi-static case. If k is between 1.0 and 10.0, the response is characterized as the intermediate case. The simulated cases of collision responses for 80 collision situations are mentioned in Figure 4 by different symbols, where the cyan symbols represent the infinite response case, the pink symbols represent the intermediate case, and the green symbols represent the quasi-static case.

For a collision situation, CCD can predict a value of ${\bar{F}}_{max}$ in the infinite response and quasi-static response regions or a range of ${\bar{F}}_{max}$ in the transition region. The case of collision can also be predicted according to the location of the point (ζ, ${\bar{F}}_{max}$) in CCD. By comparing the predicted and simulated cases of collision responses or the simulated and predicted values of ${\bar{F}}_{max}$, CCD can be investigated when the effect of the strain hardening is taken into account.

Figure 4 shows that the simulated ${\bar{F}}_{max}$ significantly depends on the strain hardening exponent n. It means that the effect of the strain hardening cannot be neglected in CCD. For the five values of the strain hardening exponent, n= 0.1, 0.2, 0.3, 0.4, and 0.5, CCD underestimates ${\bar{F}}_{max}$ from Figure 4. The error between the simulated and predicted values of ${\bar{F}}_{max}$ increases as n increases. Figure 4 also shows that CCD cannot correctly predict the cases of some simulated collisions due to the inaccurate evaluations of ${\bar{F}}_{max}$ when the effect of the strain hardening is considered. Therefore, CCD cannot characterize the collision response case and calculate the maximum collision force for the low-velocity collision considering the strain hardening effect.

### 2.3. Finite Element Analysis

**Figure 5:**
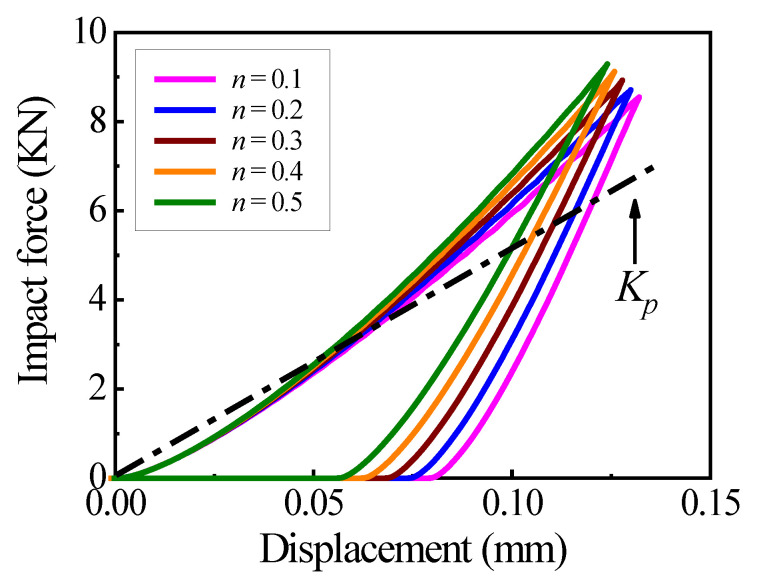
The F-δ curves for five values of n.

To investigate the reason of the inaccurate collision response case and maximum collision force by CCD, the relations of five simulated collision situations between the force F and the displacement δ are shown in Figure 5 for five values of strain hardening exponent n with the same ζ=0.01. The theoretical contact stiffness is solved by Equation (2), $K_{p}=46\;MN/m$. $K_{p}$ is constant for all of n because the strain hardening effect is not taken into account. However, from Figure 5, it can be seen that the real contact stiffness varies with n. Therefore, Equation (1) by $K_{p}$ is unreasonable and causes incorrect predictions of the response case and ${\bar{F}}_{max}$ in CCD. It means that the theoretical contact stiffness $K_{p}$ based on the theoretical contact law [20] needs revision considering the strain hardening effect.

A new contact stiffness $K_{n}$ is defined to indicate the real contact stiffness and expressed as

(6) $$K_{n}=\frac{{F_{max}}^{2}}{2S}$$

where $F_{max}$ is the maximum collision force and S is the area of F-δ curve during the loading phase from the FE simulation result. In Equation (6), $K_{n}$ is obtained from the curve of impact force $F_{max}$ versus displacement. In order to ensure that all the kinetic energy of the system is used to do work during the collision, the area S for one collision is assumed to be unchanged. The accuracy of $K_{n}$ is validated by our previous paper [36]. The new contact stiffness $K_{n}$ can be applied to replace the theoretical contact stiffness $K_{p}$ of the theoretical contact law to take the strain hardening effect into account.

## 3. Modified Collision Characterization Diagram (MCCD)

As discussed in Section 2, CCD characterizes the incorrect collision response case and calculates the inaccurate ${\bar{F}}_{max}$ for the low-velocity collision considering the strain hardening effect due to the error between the theoretical and real contact stiffness. To obtain the accurate contact stiffness, a suitable contact model is required to provide the reasonable relations of the collision force and the displacement to take the strain hardening effect into account. In this section, an expression of new contact stiffness $K_{n}$ considering the strain hardening effect is proposed to replace the theoretical contact stiffness $K_{p}$. Then, the modified collision characterization diagram (MCCD) is proposed by using the proposed $K_{n}$ to replace the theoretical contact stiffness $K_{p}$. Finally, the prediction accuracy of MCCD is validated.

### 3.1. Analytical Expression of New Contact Stiffness

To obtain the analytical expression of $K_{n}/K_{p}$ for different materials of plates struck by rigid spheres, 108 collision situations are implemented by FE simulations to cover the strain hardening exponent n from 0.1 to 0.5. The material properties and dimensions of the plate and sphere are listed in Table 3. In total, 12 cases of plates, Plate 1 to 12, with different values of $E^{*}/\sigma_{Y}$ are selected, where $E^{*}=E/\left(1-v^{2}\right)$. The range of $E^{*}/\sigma_{Y}$ represents most metallic materials. For every collision, there is a value of $K_{n}/K_{p}$, where $K_{n}$ is obtained from Equation (6) and $K_{p}$ is obtained from Equation (2).

**Figure 6:**
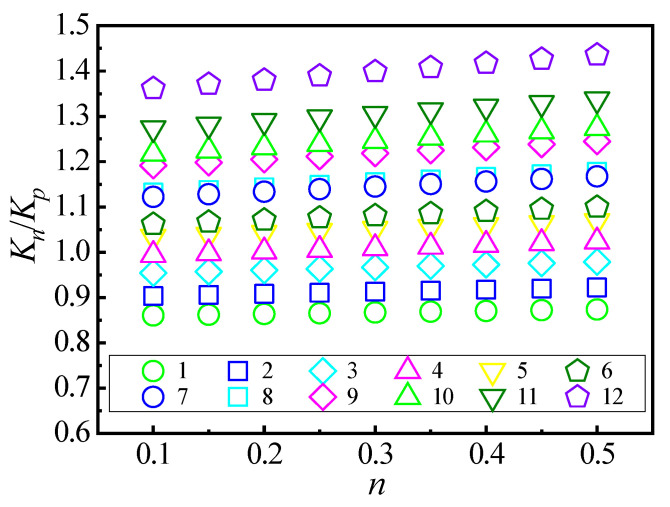
The relations between $K_{n}/K_{p}\;\mathrm{and}\;n$.

The relations between $K_{n}/K_{p}$ and n are shown in Figure 6 for different materials of plates struck by rigid spheres from the FE simulations. In Figure 6, $K_{n}/K_{p}$ increases with increasing n for a certain plate, and $K_{n}/K_{p}$ increases with increasing $E^{*}/\sigma_{Y}$ for a certain value of n. The quantitative relations between $K_{n}/K_{p}$ and n by the fitting approach from Figure 6 can be expressed as

(7) $$K_{n}/K_{p}=k,$$

(8) k=A+B·n,

where

(9) $$A=0.776+6.709\times 10^{-4}\cdot \left(E^{*}/\sigma_{Y}\right),$$

(10) $$B=0.008+2.093\times 10^{-4}\cdot \left(E^{*}/\sigma_{Y}\right).$$

From Equation (7) to Equation (10), k is dependent on n and $E^{*}/\sigma_{Y}$ and $K_{n}$ can be written as

(11) $$K_{n}=2\pi kRp_{0}.$$

The average error of $K_{n}$ is 2.07% and the maximum error is 5.86%. Therefore, Equation (11) provides a solving method of $K_{n}$ quickly and accurately considering the strain-hardening effect.

### 3.2. Construction of MCCD

By using the proposed $K_{n}$ (Equation (11)) to replace the theoretical contact stiffness $K_{p}$ (Equation (2)), a modified collision characterization diagram (MCCD) is suggested. New dimensionless maximum collision force ${\bar{F}}_{n}$, relative mobility $\zeta_{n}$, and relative stiffness $\lambda_{n}$ are applied to construct MCCD

(12) $${\bar{F}}_{n}=\frac{F_{max}}{\sqrt{2E_{0}K_{n}}},\;\zeta_{n}=\frac{\sqrt{m_{s}K_{n}}}{2c},\;\lambda_{n}=\frac{K_{st}}{K_{n}}.$$

The construction of MCCD is the same as CCD as shown in Figure 1. The infinite response region and quasi-static response boundary are obtained by using $\zeta_{n}$ and ${\bar{F}}_{n}$ to replace ζ and ${\bar{F}}_{max}$, respectively. In the quasi-static response region, the horizontal lines for different values of $\lambda_{n}$ are solved by using $\lambda_{n}$ and ${\bar{F}}_{n}$ to replace λ and ${\bar{F}}_{max}$. Because these curves and lines in MCCD have the same forms as those in CCD, three regions in MCCD are the same as in CCD (Figure 1).

### 3.3. Validations of MCCD

**Figure 7:**
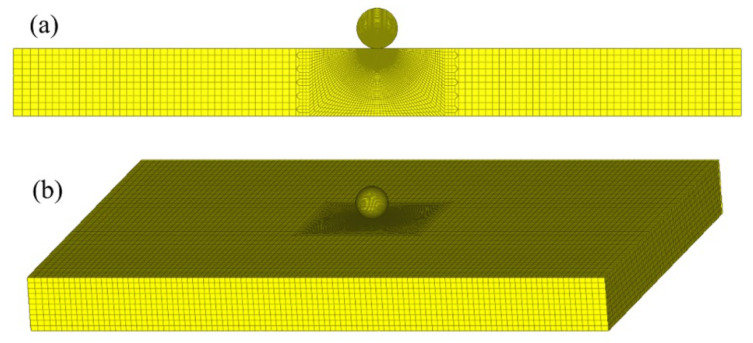
One three-dimensional FE model: (**a**) cross section and (**b**) complete model.

To validate the accuracy of MCCD by different geometries, material properties, and dimensions of plates and spheres with different constraints and collision energies, 12 collisions of circular plates stuck by rigid spheres are simulated by the axisymmetric FE models (Figure 3) and 12 collisions of rectangle plates (with the length m, width n, and thickness h) stuck by rigid spheres are simulated by the three-dimensional FE models (Figure 7). The collision energies are selected from 0.1 J to 10.0 J. The necessary information of plates and spheres are listed in Table 4 and Table 5, respectively, where C represents the clamped constraint and S represents the simply-supported constraint. For the clamped circular plate, simply-supported rectangle plate, and clamped rectangle plate, the $K_{st}$, respectively, are

$$K_{st}=\frac{16\pi D}{a^{2}},\;K_{st}=\frac{D}{0.0116mn},\;K_{st}=\frac{D}{0.0056mn},$$

where a is the radius of the circular plate, and m and n are the length and width of the rectangle plate.

**Figure 8:**
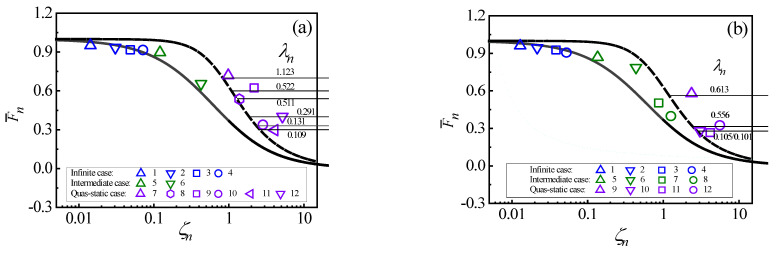
Validations of MCCD for (**a**) circular plates and (**b**) rectangle plates.

Figure 8a,b shows the corresponding points of 12 simulated collision situations of the circular plates and 12 simulated collision situations of the rectangle plates in the proposed MCCD, respectively. The cases of these collision situations are characterized by the method in Section 2.2 from the simulation results and marked various cases of responses by different symbols.

As shown in Figure 8, the response cases are characterized by MCCD correctly for the collision situations by different geometries, material properties, and dimensions of plates and spheres with different constraints and collision energies. To calculate the error between the prediction and calculation values ${\bar{F}}_{n}$, the prediction value ${\bar{F}}_{n}$ is obtained from MCCD, the calculation value ${\bar{F}}_{n}$ is obtained by Equation (11), and their error is based on the difference between the two ${\bar{F}}_{n}$. As shown in Figure 8, for the infinite response case, the prediction values ${\bar{F}}_{n}$ agree well with the calculation values ${\bar{F}}_{n}$. The maximum error of the two ${\bar{F}}_{n}$ is 5.6%. For the quasi-static case of response, the prediction values ${\bar{F}}_{n}$ agree well with the calculation values ${\bar{F}}_{n}$. The maximum error of the two ${\bar{F}}_{n}$ is 6.2%. For the intermediate case, their simulated points are in the transition region.

Therefore, MCCD can correctly be applied to characterize the case of the collision response and calculate the maximum collision force by different geometries, material properties, and dimensions of plates and spheres with different constraints and collision energies.

## 4. Conclusions

The characterization of the low-velocity collision response of a plate considering the strain hardening effect struck by the rigid sphere is investigated by the intensive FE simulations. It is found that the traditional collision characterization diagram (CCD) cannot correctly characterize the collision response case or accurately calculate the maximum collision force for the low energy collision. The reason attributes to the strain hardening effect because the real contact stiffness varies with the strain hardening exponent n. To modify CCD, a new definition of real contact stiffness $K_{n}$ is used. The universal analytical expression of $K_{n}$ is derived based on the intensive FE simulations for different materials of plates struck by rigid spheres with a wide range of strain hardening exponent n. A modified collision characterization diagram (MCCD) is then suggested by using the proposed contact stiffness $K_{n}$ to replace the theoretical contact stiffness $K_{p}$. MCCD is validated by different geometries, material properties, and dimensions of plates and spheres with different constraints and collision energies. The validations show that MCCD can give correct predictions of the collision response case and the maximum collision force. The prediction results of the low-velocity collision response of a plate have an important influence on the substantial engineering significance.

CCD can be used to predict the collision response case and calculate the maximum collision force for the collisions on the elastic–plastic plates. MCCD can correctly characterize the collision response case and accurately calculate the maximum collision force for the collisions on the elastic–plastic plates, considering the strain hardening effect, which makes up for the deficiencies of CCD. However, the effects of elastic and plastic deformations of a sphere on MCCD have not been analyzed for three types of contact: indentation, flattening, and combined types. Furthermore, potential experiments can be conducted to verify the validity of MCCD.

## Figures and Tables

**Table 1:** Dimensions and material properties.

Plate (Q345)			
a	$123\sim 640\;\left(mm\right)$	h	$8\sim 24\;\left(mm\right)$
ρ	$7800\;\left(kg/m^{3}\right)$	E	$200\;\left(GPa\right)$
$\sigma_{Y}$	$345\;\left(MPa\right)$	ν	0.3
n	0.1, 0.2, 0.3, 0.4, 0.5		
Sphere (GCr15)			
R	$7.5\;\left(mm\right)$	$m_{s}$	$0.05\sim 500\;\left(kg\right)$

**Table 2:** Sphere masses, plate sizes, and relative mobility (λ=0.3).

No.	1	2	3	4	5	6	7	8
$m_{s}\left(kg\right)$	0.05	0.2	0.5	1.0	3.0	10	20	50
$a\left(mm\right)$	640	640	640	640	640	640	640	640
$h\left(mm\right)$	24	24	24	24	24	24	24	24
ζ	0.01	0.03	0.04	0.06	0.11	0.19	0.27	0.43
No.	9	10	11	12	13	14	15	16
$m_{s}\left(kg\right)$	200	500	20	30	60	150	300	500
$a\left(mm\right)$	640	640	123	123	123	123	123	123
$h\left(mm\right)$	24	24	8	8	8	8	8	8
ζ	0.87	1.37	2.47	3.02	4.27	6.75	9.55	12.33

**Table 3:** Material properties and dimensions of plates and spheres.

Plate	1	2	3	4	5	6
E (GPa)	110	100	70	200	210	120
$\sigma_{Y}$ (MPa)	1000	600	300	700	620	320
ν	0.3	0.3	0.3	0.3	0.3	0.3
${E^{*}/\sigma }_{Y}$	121	183	256	314	372	412
n	0.1–0.5	0.1–0.5	0.1–0.5	0.1–0.5	0.1–0.5	0.1–0.5
Plate	7	8	9	10	11	12
E (GPa)	100	70	120	200	170	200
$\sigma_{Y}$ (MPa)	220	150	220	345	260	260
ν	0.3	0.3	0.3	0.3	0.3	0.3
${E^{*}/\sigma }_{Y}$	500	513	599	637	719	845
n	0.1–0.5	0.1–0.5	0.1–0.5	0.1–0.5	0.1–0.5	0.1–0.5
R=7.5mm $,\;m_{s}=0.5$ kg, a=640 mm, h=24 $\;\mathrm{mm},\;\rho =7800\;kg/m^{3}$						

**Table 4:** Twelve simulated collision situations of circular plates.

No.	1	2	3	4	5	6	7	8	9	10	11	12
$a\left(mm\right)$	380	420	200	250	600	640	200	320	150	160	320	120
$h\left(mm\right)$	32	32	32	32	48	48	24	24	12	12	12	8
$R\left(mm\right)$	10	10	10	10	15	15	15	15	7.5	15	15	10
$m_{s}\left(kg\right)$	0.05	0.2	3.5	8	5	50	16	10	80	50	100	220
$E\left(GPa\right)$	70	200	210	120	70	100	120	200	200	200	70	110
$\sigma_{Y}\left(MPa\right)$	300	700	620	320	150	220	220	345	345	345	300	620
ν	0.3	0.3	0.35	0.35	0.3	0.3	0.35	0.3	0.3	0.3	0.3	0.35
n	0.1	0.2	0.3	0.4	0.5	0.15	0.25	0.35	0.45	0.1	0.2	0.3
$E_{0}\left(J\right)$	0.1	0.5	1.0	2.0	3.0	4.0	5.0	6.0	7.0	8.0	9.0	10.0
Constrain	C	C	S	S	S	S	S	S	C	C	S	S
$\zeta_{n}$	0.015	0.038	0.052	0.075	0.136	0.452	0.926	1.322	2.202	2.721	4.157	5.213
$\lambda_{n}$	0.658	0.586	1.578	1.215	0.643	0.498	1.123	0.511	0.522	0.131	0.109	0.291

**Table 5:** Twelve simulated collision situations of rectangle plates.

No.	1	2	3	4	5	6	7	8	9	10	11	12
$m\left(mm\right)$	400	500	600	640	800	800	640	640	480	480	640	600
$n\left(mm\right)$	400	500	500	480	720	720	480	480	240	240	480	500
$h\left(mm\right)$	25	25	50	50	25	25	12	12	12	12	10	10
$R\left(mm\right)$	8	16	8	8	8	16	8	16	10	32	16	8
$m_{s}\left(kg\right)$	0.05	0.2	0.3	0.6	20	160	100	200	125	180	200	250
$E\left(GPa\right)$	70	200	210	120	70	100	120	200	200	200	70	110
$\sigma_{Y}\left(MPa\right)$	300	700	620	320	150	220	220	345	345	345	300	620
$\nu_{p}$	0.3	0.3	0.35	0.35	0.3	0.3	0.35	0.3	0.3	0.3	0.3	0.35
n	0.1	0.15	0.2	0.25	0.3	0.35	0.4	0.45	0.5	0.15	0.25	0.35
$E_{0}\left(J\right)$	0.1	0.5	1.0	2.0	3.0	4.0	5.0	6.0	7.0	8.0	9.0	10.0
Constrain	C	C	C	S	S	S	C	C	C	C	S	S
$\zeta_{n}$	0.016	0.026	0.041	0.059	0.155	0.476	0.812	1.321	2.411	3.111	4.278	6.123
$\lambda_{n}$	4.513	8.469	3.889	11.39	0.612	0.886	0.398	0.166	0.556	0.613	0.101	0.105

## Data Availability

The original contributions presented in this study are included in the article. Further inquiries can be directed to the corresponding author.
